# Diverting the Ureters and Removing the Bladder

**Published:** 1888-04

**Authors:** 


### DIVERTING THE URETERS AND REMOVING THE BLADDER.

The futility of partial operative measures in cancer of the bladder seems to be established. In Vol. XVI, of *St. Thomas' Hospital Reports*, Mr. WALTER EDMUNDS and Mr. CHARLES A. BALLANCE have a contribution on the "Best Method of Diverting the Ureters and Removing the Bladder, as shown by experiments upon the Cadaver."

The authors think that malignant disease of the bladder should be looked upon in exactly the same way as cancer of other organs or parts of the body that are not essential to life; in other words, a radical removal of the diseased organ should be undertaken in suitable cases, rather than a scraping, nibbling, or incomplete operation that is certain to be followed by a rapid recurrence of the growth. They hold that as soon as the diagnosis of cancer is certain, by exploration or otherwise, a much more complete operation should be undertaken than is at present in vogue. At best, they believe, the bladder is merely an organ of convenience, and one by no means necessary to life. A man would be better off without a bladder than with one that harbored a cancerous graft. Not a few persons go about comfortably with one urinary sinus; why should not others go about with two? Moreover, when the ureters are diverted the bladder no longer has any active function to perform—it exists simply passively in the body, and its blood supply diminishes in obedience to the lessened physiological activity of the parts; so that, in a given case, if it were decided to divert the ureters and leave the bladder alone, the surgeon might well anticipate a general shrinkage of the parts and a temporary arrest of the disease, or a slower growth of it, as happens in cancer of the rectum when the fæces are diverted by complete division of the colon. Furthermore, it may be noted that in cancer of the bladder, lymphatic gland infection seems to occur only at a later stage, a fact that tells in favor of radical treatment.

The method proposed and recommended is as follows: 1. The first step is to divide and bring out the ureters. They had best be treated on separate occasions, and thus two preliminary operations would be required before any interference with the bladder is attempted.

The ureter can be reached by an incision through the front of the abdomen, somewhat similar to that for ligaturing the common iliac artery, but the most convienent position for the incision, without allowing for variations in the size of different abdomens, is as follows:

The incision is curved with the convexity outward; it commences vertically above the spine of the pubes, at the level of the junction of the middle and lower thirds of an imaginary line connecting the umbilicus and symphysis pubis. From this point it extends outward and slightly upward to within an inch and a half of the anterior superior spine, then upward, and lastly upward and inward in the direction of the umbilicus, until a point is reached vertically above the commencement of the incision. The abdominal parietes external to the rectus are then divided until the peritoneum is reached. The skin and fascia over the rectus corresponding to the incision directions are cut through to give room, but the muscle and its sheath should be left intact. The peritoneum can then be reflected from the iliac fossa until the ureter is seen attached to the peritoneum in the region of the common iliac artery. It adheres by means of some areolar tissue to the peritoneum. If this be not borne in mind the operator will look in vain for the ureter.

When found, the ureter is followed down toward the bladder, and no difficulty will be experienced in applying a double ligature of silk to it at a distance of from a half to an inch from the spot where it enters the bladder wall. It is then to be divided, and the proximal end brought up into the iliac fossa. Next, the lower end of the upper portion of the ureter must be brought to the surface of the body, and this is best effected through a *separate incision*. The position of the opening should be immediately over and about half an inch behind the crest of the ilium—a point that is at least four inches from the large incision. The ureter should be passed through an opening made here, which should be just large enough to admit it, and should be secured by fine silk sutures, half an inch or more of the tube being caused to project beyond the surface of the skin. Care must be taken to separate the ureter from its bed for a short distance above the brim of the pelvis, so that it may pass to its new outlet by a gentle curve, and not be bent near the iliac artery at a right angle. It is necessary, too, that the ureter be caused to lie in contact with the posterior muscular boundary of the abdomen after it has been displaced from its old position, so that the parietal peritoneum and the intestines may readily fall back again against the quadratus and the psoas, and into the iliac fossa.

It need hardly be added that great care should be taken to asepticise the upper end of the distal portion of the ureter, that the lesser wound should be securely sealed, so that it may not be the cause, by inward leakage, of infection to the greater, and that the two wounds should be entirely shut off from each other—the larger wound being kept antiseptic and free from the contamination of the smaller at which the urine would flow from the ureter.

*Excision of the Bladder.*—At the proper time, after diversion of the ureters has been done, the bladder may be excised by the supra-pubic method. A vertical incision is made from the symphysis to about half way up to the umbilicus, the lower border of the peritoneum is found, and the membrane is then reflected off the bladder. This is easily done as far as the entrance to the ureters by means of the fingers, and by the aid of a pair of curved, blunt-pointed scissors to snip off any areolar tissue that cannot be overcome by gentle manipulations with the fingers. The anterior lateral and posterior walls of the bladder are now free from all attachments, and can be removed at the will of the operator. It is not desirable to take away more than the mucous membrane in the region of the trigone in consequence of the vascular and other important relations of the base of the bladder and prostate. Fortunately, carcinoma is limited at first to the mucous membrane, and this can easily be peeled off at the base of the bladder from the muscular coat.

As the ureters extend to the surface of the skin, stricture of one or both of the apertures of exit would not be likely to occur, so that the patients subjected to the plan of treatment above described would be measurably free from the dangers of hydronephrosis and other affections that ensue when urine is directed away from the ureter in other ways and under other circumstances, and that are the common consequences of the obstruction that follows prolonged cystitis, *e.g.*, in cases of extrophy of the bladder and chronic untreated urethral stricture.

For placing a double ligature upon the ureter deep in the pelvic cavity good light is necessary; electric light or strong bright daylight should be used, and directed into the wound by hand mirrors.

The authors call special attention to two points: 1. The good results that would probably follow complete diversion of the urine from the bladder in cases of cancer, quite independent of any further operation upon the organ; under this circumstance a partial operation even would probably be followed by better results. 2. Double urinary fistulæ and their effects upon the kidney should be thoroughly studied upon animals before the operation is attempted upon a patient.

